# Movement trajectories as a window into the dynamics of emerging neural representations

**DOI:** 10.1038/s41598-024-62135-7

**Published:** 2024-05-20

**Authors:** Roger Koenig-Robert, Genevieve L. Quek, Tijl Grootswagers, Manuel Varlet

**Affiliations:** 1 The MARCS Institute for Brain, Behaviour and Development, Western Sydney University, Penrith, NSW 2751 Australia; 2 School of Psychology, University of New South Wales, Sydney, NSW Australia; 3 School of Computer, Data and Mathematical Sciences, Western Sydney University, Penrith, NSW 2751 Australia; 4 School of Psychology, Western Sydney University, Sydney, NSW 2751 Australia

**Keywords:** Cognitive neuroscience, Computational neuroscience, Sensory processing

## Abstract

The rapid transformation of sensory inputs into meaningful neural representations is critical to adaptive human behaviour. While non-invasive neuroimaging methods are the de-facto method for investigating neural representations, they remain expensive, not widely available, time-consuming, and restrictive. Here we show that movement trajectories can be used to measure emerging neural representations with fine temporal resolution. By combining online computer mouse-tracking and publicly available neuroimaging data via representational similarity analysis (RSA), we show that movement trajectories track the unfolding of stimulus- and category-wise neural representations along key dimensions of the human visual system. We demonstrate that time-resolved representational structures derived from movement trajectories overlap with those derived from M/EEG (albeit delayed) and those derived from fMRI in functionally-relevant brain areas. Our findings highlight the richness of movement trajectories and the power of the RSA framework to reveal and compare their information content, opening new avenues to better understand human perception.

## Introduction

The human brain’s capacity for transforming visual input into meaningful mental representations enables our species’ adaptive behaviour in complex and continuously changing environments. These representations propagate rapidly along the visual hierarchy, enabling complex objects, like a face or a dog, to be categorised within just a few hundred milliseconds after light hits the retina. Early neural representations and their associated brain regions mainly appear to encode perceptual features of visual stimuli, while later representations in higher-level regions capture conceptual and semantic associations^1–3^. The processes supporting such remarkably efficient object recognition develop rapidly during infancy, maturing to become highly automatic and consistent across individual brains and task conditions^4–6^. While neuroimaging methods have been central to efforts to understand such visual processes, here we show that *human movement trajectories* are a powerful complementary means of gaining insight into the temporal dynamics of unfolding neural representations.

Computer mouse-tracking movement trajectories are a relatively recent development in behavioural research^7–10^, wherein the cursor is continuously tracked while an observer selects between response options. This low-cost and widely available method has been especially useful for indexing non-explicit processes such as self-control, emotion, ambivalence, moral and subliminal cognition^11–15^*.* Unlike discrete measures such as button-presses, movement trajectories have been argued to provide a window into the temporal dynamics of cognitive processes, revealing the emergence and duration of cognitive phenomena^7,10,16–19^*,* rather than only the end-point of decisional and motoric processes. Yet, the extent to which movement trajectories index the *continuous unfolding* of cognitive processes, such as the transformation of visual inputs into meaningful neural representations, remains controversial^7,10,20^. It is still highly debated whether movements, especially those performed under time constraints, can be continuously modified by cognitive processes once their execution has started. For instance, studies have suggested that certain changes in trajectories may not be visually informed^21^, that early visual perception might not be penetrable by cognition^22^, that the variability of movement outcomes might be mainly related to preparatory neural activity^23,24^, and that only single motor plans (i.e., a single choice, instead of competition among choices) would be represented in the motor cortex^25^, thus challenging the hypothesis that the timecourse of emerging neural representations can be captured via movement trajectories.

In this study, we leveraged representational similarity analysis (RSA^26,27^)—which models information carried by measurement units rather than activation in those units themselves^6,28,29^—to reveal the time-varying representational structures contained in movement trajectories and demonstrate their correspondence with those obtained from neuroimaging data. By virtue of their automaticity, rapid timecourse, and robustness to task demands, object representations are an ideal scaffold for probing the capacity of movement trajectories to meaningfully capture unfolding perceptual processes. We focus on two key organising principles in object vision—namely, faces *vs.* objects, and animals *vs.* inanimate objects—critical for social interactions and survival^30^. Stimuli in these classes reliably evoke dissociable neural representations whose timecourse and underlying brain regions are well-characterised. At the same time, a subset of ambiguous or ‘lookalike’ stimuli challenge our robust object categorisation abilities, for example, inanimate objects that have face-like features (i.e., illusory faces or *pareidolia*). Such stimuli provide an intriguing glimpse into the whorl of perceptual and conceptual processes that converge into high-level categorisations, and are ideally suited to test the sensitivity of movement trajectories as they represent an intermediate class whose neural representations dissociate from those of unambiguous category members in predictable ways^31–34^.

**Figure 1:**
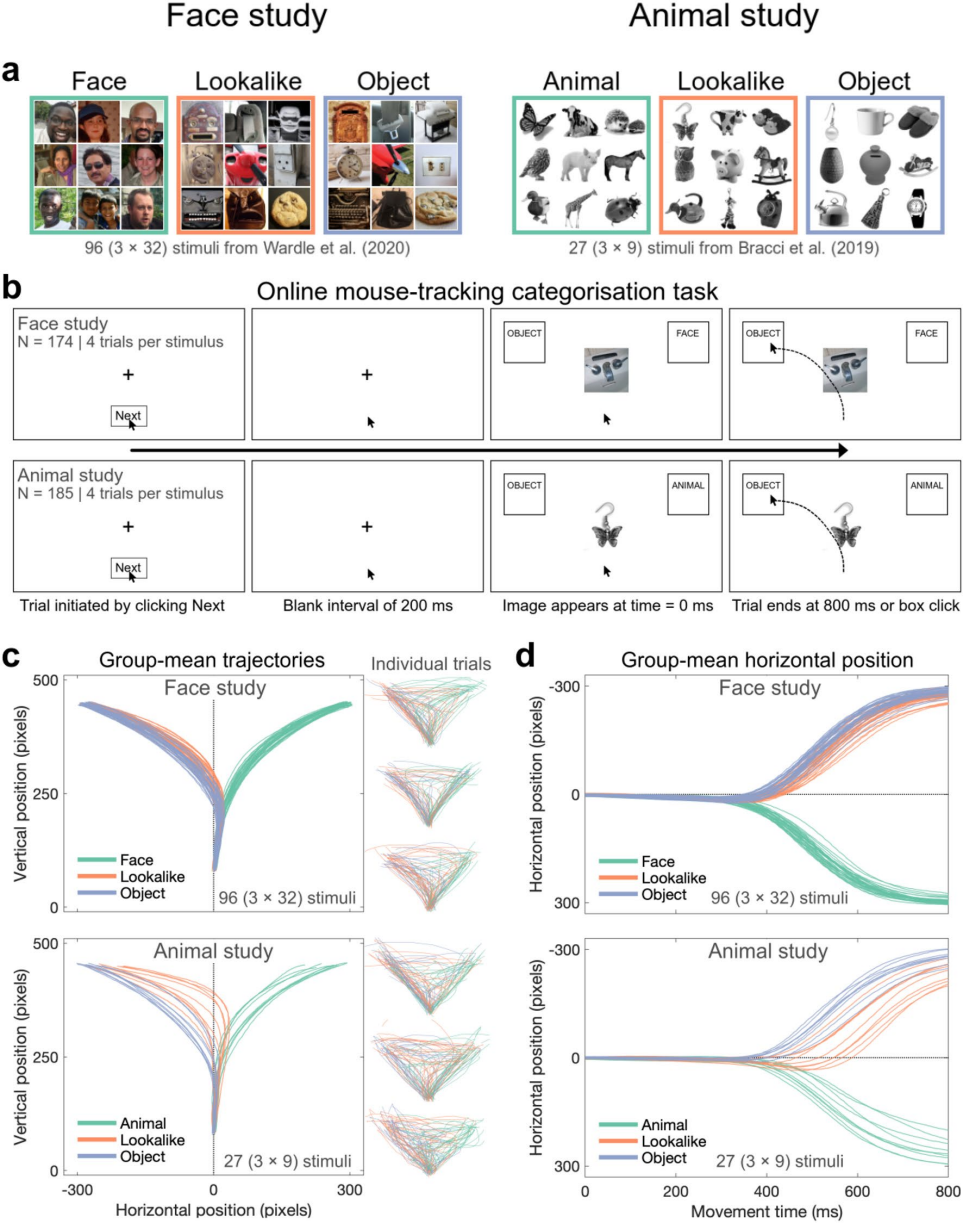
Mouse-tracking paradigm overview. (**a**) Stimuli. The Face Study used 32 face, 32 object, and 32 matched lookalike images (illusory faces) as in^33^. The Animal Study used 9 animal, 9 object, and 9 matched lookalike images (objects that look like animals) as in^31^ and^32^. In both studies, we added images to equate the number of exemplars in each response category (e.g., 32 extra face stimuli in the Face Study); these were excluded from the analysis. (**b**) Categorisation Task Sequence. The stimulus appeared 200 ms after the participant clicked on the ‘Next’ button to initiate the trial. Participants had 800 ms from stimulus onset to categorise the image by moving the cursor to one of the two response boxes. The cursor position was recorded continuously, resulting in a categorisation movement trajectory on every trial. Response box positions were swapped halfway through the task to minimise left/right bias. In both studies, we instructed participants to categorise lookalike stimuli as objects (i.e., the ground truth). (**c**) Group-mean cursor trajectories for individual stimuli. At right: Individual trial data for three example participants in each study. (**d**) Mean horizontal cursor position for each stimulus, shown as a function of time. Since the horizontal axis was the relevant decision axis in our task (e.g., go left for OBJECT, go right for FACE/ANIMAL), time-varying horizontal position served as our dependent variable. For each image, we averaged horizontal position at each timepoint first within and then across participants in each study, and used these summary scores for further analyses.

In two online experiments, observers categorised both unambiguous (Faces/Objects in Study 1, Animals/Objects in Study 2) and ambiguous (Lookalike) category exemplars by moving the cursor to click on an upper left or right response box, as depicted in Fig. 1. We used stimuli from previously published studies^31–33^ with publicly available neuroimaging data (M/EEG and fMRI). We show that time-varying representational structures in movement trajectories, as reflected in timepoint-by-timepoint representational dissimilarity matrices (RDMs), provide a meaningful, non-contemporaneous index of dynamically evolving neural representations in the human brain, which are not fully captured in end-point behavioural measures (e.g., explicit face ratings and movement execution times). By comparing movement trajectories over time to both theoretical models and M/EEG neuroimaging representational structures, we found strong evidence that movement trajectories indeed capture (with a delay) image-wise and category-wise differences evident from the earliest stages of visual processing through to stable semantic category structures. Furthermore, the observed representational overlap between movement trajectory and time-resolved M/EEG data was predicted by representational structures derived from neural activity in functionally-relevant brain areas as measured by fMRI. This work demonstrates that movement trajectories can serve as a valuable tool for capturing the temporal dynamics of unfolding representational structures, and that movement trajectories can provide insights that complement and extend current behavioural and neuroimaging methods.

## Results

We analysed mouse trajectory data from 174 online observers^35^ as they performed a face *vs.* object categorisation task on the stimuli from^33^ and 185 online observers as they performed an animal *vs.* object categorisation task on the stimuli from^31^ and^32^ (Fig. 1a). Each trial automatically terminated 800 ms after stimulus presentation, or when the participant clicked on a response box. The short response deadline encouraged participants to begin their movement immediately after clicking the ‘Next’ button to initiate the trial, even though the stimulus itself did not appear until 200 ms after the trial was initiated (Fig. 1b). Despite this time pressure, analysis of mouse trajectory endpoints showed that participants were accurate in categorising all three image categories (83.62, 80.91 and 83.13% for face, lookalike, and object stimuli in the Face Study, respectively; and 83.52, 77.90 and 84.85% for animal, lookalike, and object stimuli in the Animal Study, respectively). Since our brief online experiment allowed for only 4 categorisations of each stimulus, we pooled the online categorisation data^36,37^ by computing the group-mean movement trajectory for each image, obtained by averaging all trajectories for a given image firstly within each participant, and then across all participants.

Group-mean movement trajectories for each image (Fig. 1c) contained relevant information about stimulus category from a very early point (horizontal cursor position data given in Fig. 1d). Trajectories showed a slight initial tendency towards responding ‘FACE’ and ‘ANIMAL’, which could be caused by several factors (e.g., a bias towards animate stimuli^38,39^ that affects participants’ interpretation of the task—treating it akin to a Go/No-Go face/animal task). Importantly, and despite this initial bias, trajectories for each category rapidly diverge from a very early stage of the movement. Specifically, trajectories for exemplars in the two unambiguous categories diverged from each other soonest (i.e., approximately 325 ms in both studies). In contrast, trajectories for ambiguous (Lookalike) stimuli diverged from their ‘confusable’ categories comparatively later (around 400 ms), with this effect being more accentuated in the Animal Study. These qualitative patterns suggest that cursor movements were not ballistic in nature, but rather dynamically updated (i.e., at different points during the movement execution) to incorporate additional evidence supporting final categorisation response.

### Representational structures in movement trajectories and M/EEG data

**Figure 2:**
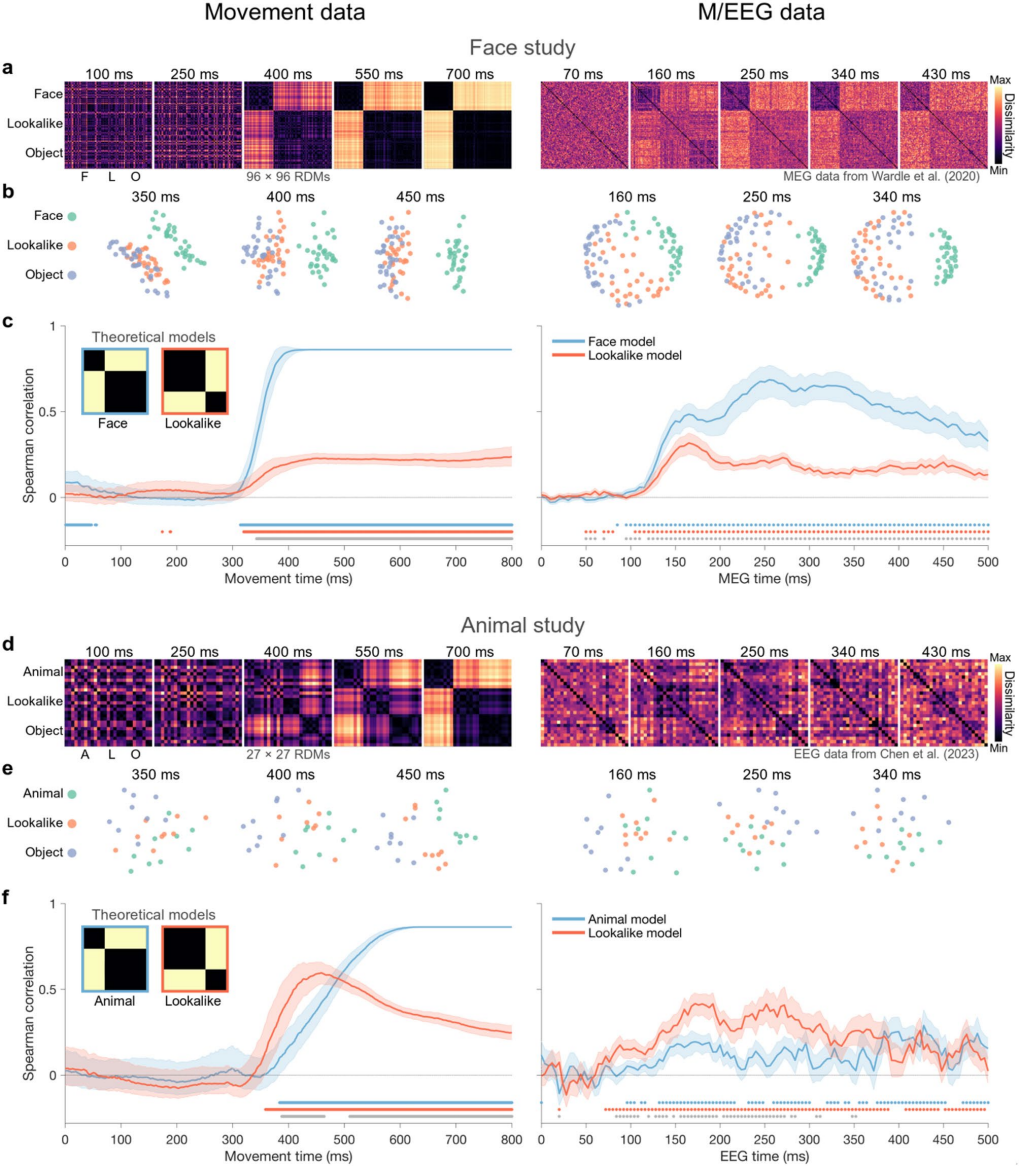
Movement and M/EEG representational structures. The left column represents movement trajectory data (i.e., horizontal position) from 0 to 800 ms after stimulus presentation; the right column represents pre-existing M/EEG data from 0 to 500 ms after stimulus presentation. (**a**,**d**) Selected timepoints of the RDM series derived from Movement (left) and M/EEG (right) data. RDMs reflect rank-ordered pairwise dissimilarity between images, i.e., pairwise horizontal position differences in movement data, pairwise 1-correlation values between MEG activation patterns, and pairwise decoding accuracies for EEG data. (**b**,**e**) Associated two-dimensional embeddings that reflect the degree of similarity between all stimuli at selected timepoints, computed using multidimensional scaling. For the movement data, embeddings were based on RDMs averaged across a 50 ms window centred on the indicated timepoint to maximise multidimensional variance. (**c**,**f**) Correlation with theoretical models. Correlation between the theoretical Face/Animal (task) model and Lookalike model (inset) and each RDM series. Shaded regions show 5th and 95th percentile correlation values from 10,000 permutations with random subject resampling with replacement to estimate inter-subject variability. Coloured dots indicate one-tailed significant differences from 0 for each model (*p* < 0.05); grey dots indicate two-tailed significant differences from 0 for between model differences (*p* < 0.05). For each study, the dominant model in the neural data mirrors that found in our trajectory data.

We used timepoint-by-timepoint representational dissimilarity matrices (RDMs)^26^ derived from movement trajectory data to reveal the time-resolved representational structure of observers’ categorisation movements. At each 1 ms timepoint, we calculated pairwise differences in mean horizontal cursor position for each pair of stimuli. We used the horizontal cursor position (as opposed to both horizontal and vertical coordinates, or time derivatives of these) as it was the critical dimension and most direct descriptor in our categorisation task (i.e., go left for OBJECT, go right for FACE/ANIMAL; see “Methods” for details). Movement RDMs for both studies evidenced clear task-related representational structures that evolved over the course of the movement. Overall dissimilarity between face/animal stimuli and other stimuli (lookalike and object) increased to reach maximum dissimilarity towards the end of the trials. More importantly, movement RDMs revealed early perceptual differences between lookalike stimuli and unambiguous object stimuli; lookalike trajectories stayed closer to trajectories of the category they could be confused for (i.e., Face; Animal) for longer. Two-dimensional embeddings shown in Fig. 2b,e highlight this greater representational overlap between lookalike and face/animal stimuli compared to object and face/animal stimuli, with this effect being stronger in the Animal Study.

To quantify the effects of stimulus category on movement trajectories, we correlated the RDM series derived from horizontal cursor position (Fig. 1a) with two theoretical models (Fig. 1c, inset). The first was a ‘ground truth’ Face/Animal model representing the actual categorisation task participants had to perform (where lookalike stimuli should be categorised as objects). The second was a Lookalike model which enabled us to test the hypothesis that lookalike stimuli are perceived similarly to their confusable category (i.e., Face/Animal). Correlation with the Face model in the Face Study increased sharply after 300 ms, reaching asymptote at approximately 400 ms, whereas the correlation with the Animal model in the Animal Study increased more gradually from 300 ms and reached asymptote at approx. 600 ms. Movement RDMs in both studies also correlated with the Lookalike model, confirming that movement trajectories effectively captured the perceptual ambiguity of lookalike stimuli. This correlation was stronger in the Animal Study and started even earlier than the correlation with the Animal model (see Fig. 2c,f, left column).

Notably, the model correlations for our trajectory data were highly similar to those found for the M/EEG data for these stimuli (Fig. 2c,f, right column). Both MEG RDMs in the Face Study and EEG RDMs in the Animal Study showed that the Lookalike model was a relevant predictor of neural responses, indicating that lookalike stimuli evoked neural responses with transient categorical ambiguity. The pattern of relative model dominance across the two studies suggests this effect was more pronounced in the Animal Study, where the Lookalike model was the dominant predictor for much of the neural response. In contrast, in the Face Study, the Face model predicted the MEG RDMs better than the Lookalike model. In line with the movement RDMs, correlation with the Animal model in the Animal Study also appeared slightly delayed compared to the Lookalike model, whereas correlations with the two models in the Face Study seem to arise at the same time. A relevant point to note is that the MEG data from^33^ were collected in the absence of an active categorisation task. As such, the strong concordance with the Face model in this data highlights the largely automatic nature of the neural processes supporting face/object categorisation. EEG data from^32^ were collected while participants performed either an animal *vs.* object task or a lookalike *vs.* object classification task. The EEG RDMs we use here are based on data from both tasks, since the RDM series varied only slightly as a function of task condition (see^32^). In sum, regardless of the task, movement trajectories and time-varying neural responses showed remarkable similarities in the ways categorical patterns emerge. We next quantified these similarities by directly comparing their representational structures.

### Representational similarity between movement trajectories and M/EEG data

We directly tested the evolving representational overlap between movement and M/EEG RDMs by performing a time-time correlation between the two RDM series that took account of their different timescales. It is important to note that correlations between movement trajectories and M/EEG data are not due to the similarities in their signal dynamics (i.e., signal shape or order) but rather to the temporally resolved differences between pairwise trajectories and M/EEG signals.

**Figure 3:**
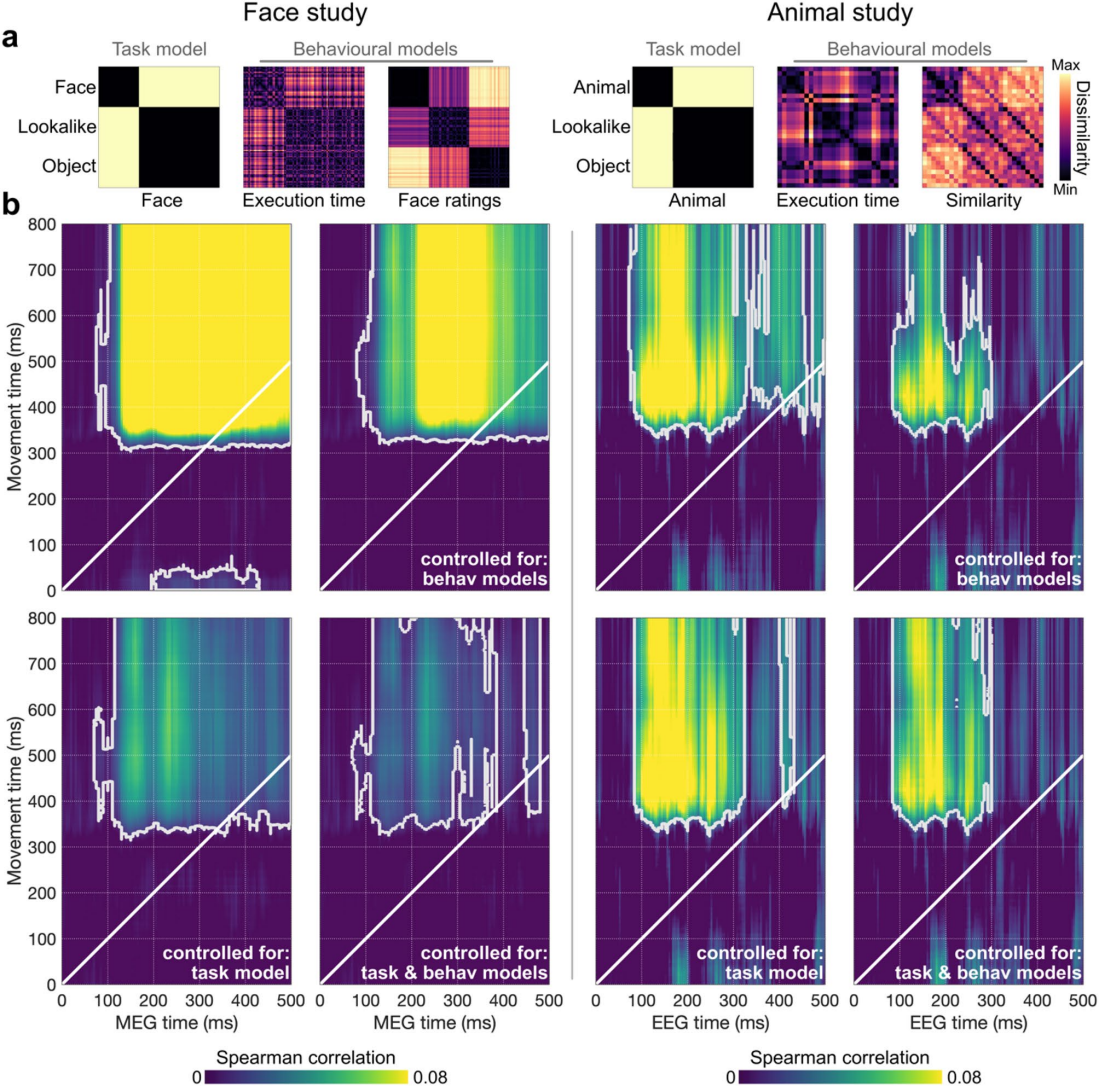
Representational overlap between movement trajectories and M/EEG data. (**a**) Control Model RDMs reflecting (*i*) the categorisation task, (*ii*) overall movement execution time, and (*iii*) existing behavioural data for the stimuli in each study, taken from^33^ (face ratings) and^31^ (stimulus similarity). (**b**) Time-time correlations between movement and M/EEG RDM series. White outlines denote significant correlation clusters based on 10,000 permutations. For each study, we show the raw time-time correlation values (top left), correlation values controlled for the behavioural models (top right), correlation values controlled for the task model (bottom left), and correlation values controlled for both the task and behavioural models (bottom right). In both studies, we find representational overlap between movement trajectories and M/EEG responses that is not explained by these control models.

Figure 3 shows significant clusters of correlation between movement and M/EEG RDMs which appear shifted upwards from the diagonal (which represents identical movement and M/EEG times). This indicates that representations reflected in movement trajectories lagged in time compared to those captured by M/EEG data, with significant correlations starting from about 70 ms and 300–350 ms for M/EEG and movement, respectively. Importantly, these clusters of significant correlation persisted even when we controlled for correlations driven by the Face/Animal model which represents the categorisation task performed by our participants.

Moreover, we found that clusters of significant correlation also persisted when controlling for representational structures derived from other conventional behavioural measures. For the Face Study, we used the face rating RDM from^33^ (Fig. 3a). These face ratings (*face-likeness* of each image on a scale from 0 to 10) were completed by independent online observers (*N* = 20), see “Methods” and^33^ for details. In the Animal Study, we used similarity judgements from^31^ (Fig. 3a). Participants (*N* = 17) in this study were instructed to ‘arrange objects according to how similar they are’ and were free to choose the dimensions they considered the most relevant, see “Methods” and^31^ for details. To confirm the complementarity of movement trajectories to conventional measures of processing/response speed, we included a movement execution time RDM as an additional control model—computed as pairwise differences in the time at which participants’ cursor reached the response box. These behavioural models only partially explained the time-time representational correlations between movement and M/EEG data, with significant clusters of correlation remaining after controlling for these models, particularly within the first hundred milliseconds of M/EEG data after stimulus presentation. This clearly demonstrates the added value that movement trajectories can offer over discrete behavioural measures (e.g., ratings, reaction time) in terms of indexing early perceptual processing.

### Image-wise representational similarity between movement trajectories and M/EEG data

**Figure 4:**
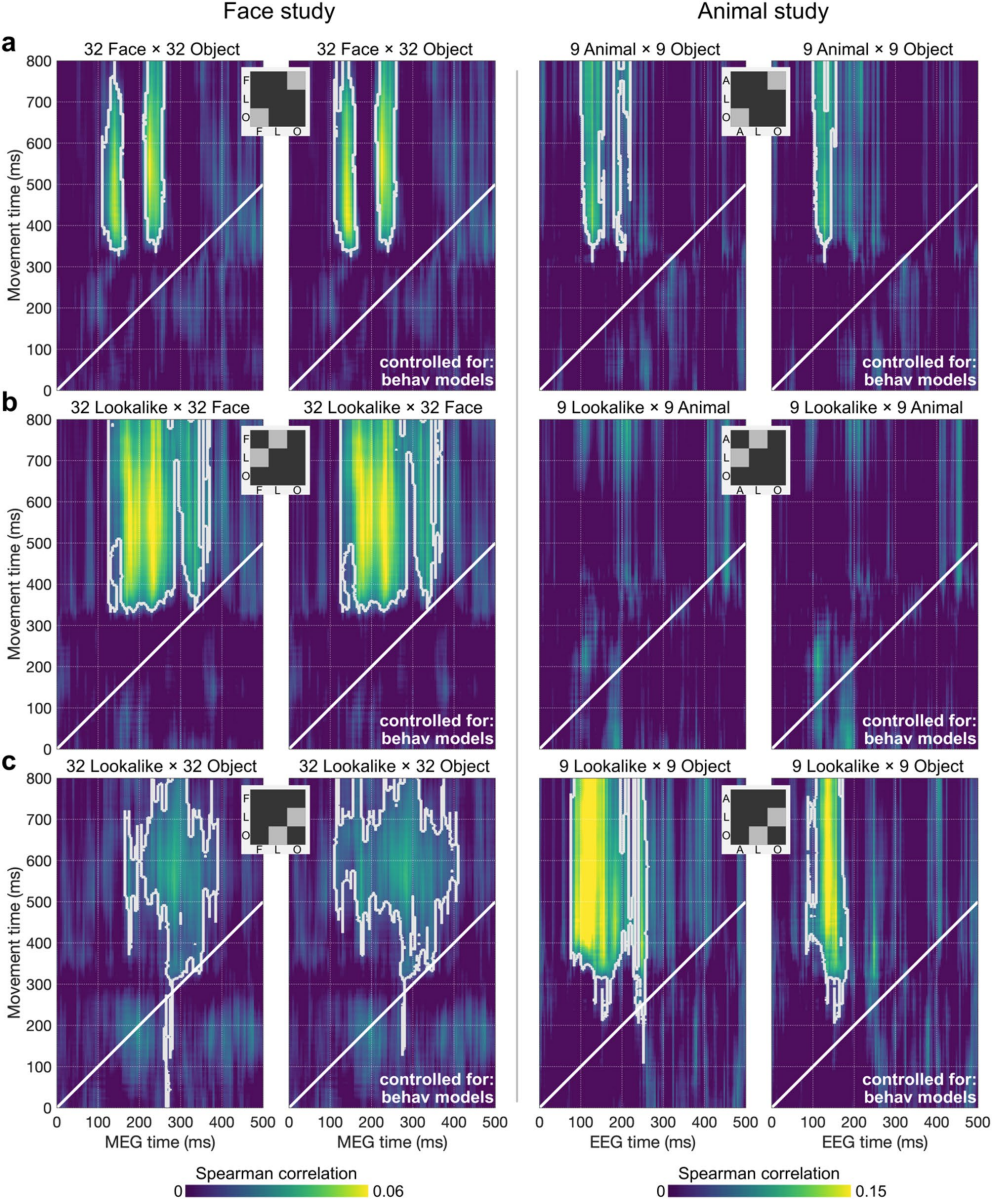
Time-time correlations between movement and M/EEG data for specific category-pairings subsumed within the full RDM. (**a**) Time-time correlation restricted to the unambiguous stimulus contrast (i.e., Face/Animal × Object), where movement data show representational overlap with early M/EEG representations (from about 100 ms) that capture the rapid categorisation of these unambiguous category members. (**b**,**c**) Time-time correlation restricted to the ambiguous stimulus contrasts: Lookalike × Face/Animal and Lookalike × Object. Here movement data show representational overlap with comparatively later M/EEG representations, suggesting that lookalike stimuli are separated from unambiguous category members more slowly. Controlling for behavioural models leaves time-time correlations for the various subsets in both studies largely unchanged, highlighting the capacity of movement trajectories to capture subtle representational differences at the level of individual images. White outlines denote significant correlation clusters based on 10,000 permutations.

We next applied our time-time correlation approach to specific category-pairings within the full RDM (i.e., RDM subsets, Fig. 4), with the goal of examining representational overlap between movement and M/EEG data at the level of individual images. These subsets represent the intersection of two categories at a time (e.g., Lookalike × Object), thus capturing image-wise representational similarity correlations between movement and M/EEG RDMs that cannot be accounted for by broad representational differences between categories (as is the case in the full RDM). In the Face Study, significant correlation clusters were evident for all three RDM subsets—including the unambiguous stimulus contrast (Face × Object), and both contrasts including the ambiguous stimuli (i.e., Lookalike × Face and Lookalike × Object, see left column in Fig. 4). In the Animal Study, significant clusters were found for the unambiguous stimulus contrast (Animal × Object) and for the ambiguous contrast Lookalike × Object, as seen in Fig. 4 (right column). No significant cluster was found for the Lookalike × Animal subset—an outcome which could be due to insufficient data when reducing RDMs to 9 × 9 image subsets and/or higher similarity in the neural responses to animal and lookalike stimuli leaving insufficient variance (see Fig. 2 and decoding accuracy in^32^). Note that time-time correlations for the various subsets in both studies were virtually unchanged by controlling for the behavioural models (Fig. 4, second and fourth columns), demonstrating that movement trajectories are uniquely suited for capturing subtle image-level representational differences during the early stages of visual processing.

Interestingly, we found interpretable differences in the timing of representational overlap between movement and M/EEG data across the different subsets in both studies. In the Face Study (Fig. 4, left columns), time-time correlation for the unambiguous contrast (Face × Object) revealed overlap between our mouse trajectory data and early MEG representations (from about 100 ms) reflecting rapid and robust neural distinction between face and object stimuli. In contrast, for the two ambiguous subsets (Lookalike × Face and Lookalike × Object), representational structures in trajectory data correlated with comparatively later MEG representational structures. A similar pattern was observed in the Animal Study (Fig. 4, right columns), with movement representational structures overlapping with early EEG representational structures (from about 100 ms) for the unambiguous contrast (Animal × Object). For the ambiguous subset Lookalike × Object, representational correlation extended to later EEG representational structures, but notably, also exhibited an early onset in EEG time from about 100 ms, likely capturing the strong degree of resemblance between the Lookalike stimuli and their confusable category (Animal) in this study (see Lookalike model correlations with EEG data in Fig. 2f).

### Region-specific contribution to representational overlap between movement trajectories and M/EEG data

**Figure 5:**
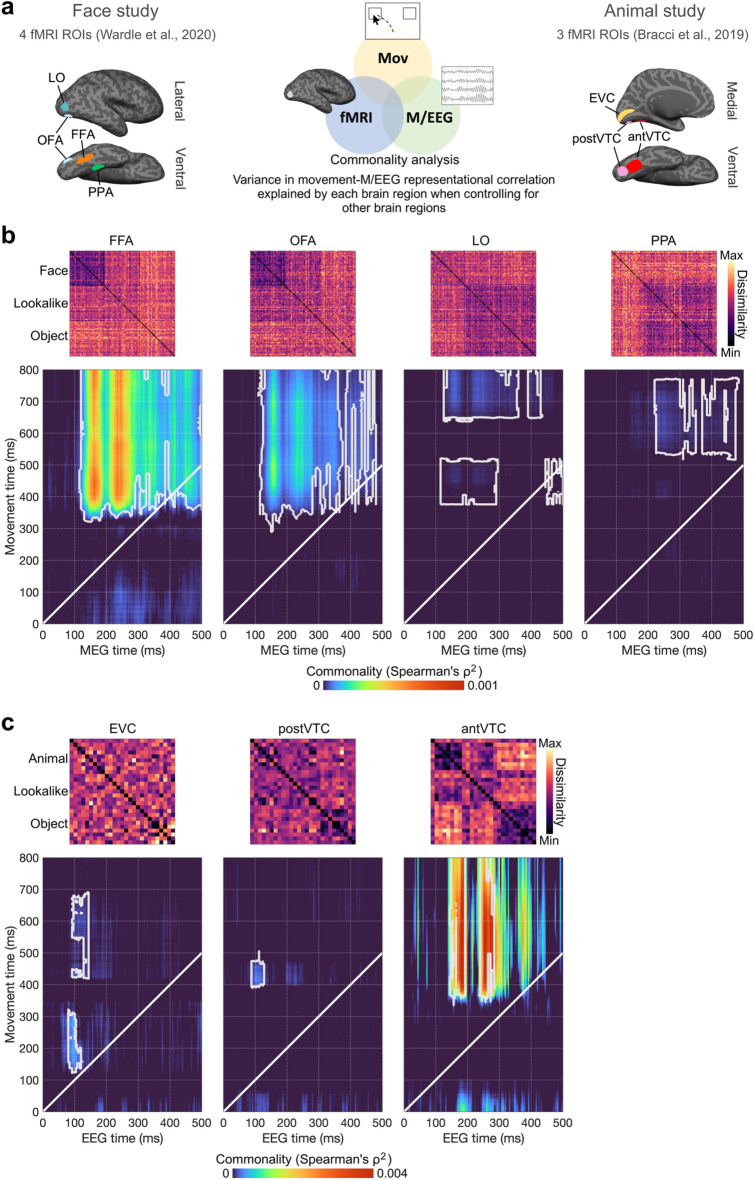
Movement-M/EEG-fMRI Commonality analysis. (**a**) Overview & ROIs. Commonality analysis probes the degree to which RDMs derived from the pre-existing fMRI data in different brain areas can uniquely account for the shared representational structure observed between the full RDM series for movement and M/EEG data (controlling for task). There were four ROIs in the Face Study (FFA, OFA, LO, PPA) and three ROIs in the Animal Study (EVC, posterior VTC, anterior VTC). (**b**) fMRI ROI commonality in the Face Study. The shared representational structure between trajectory and MEG data was most concordant with the RDM derived from BOLD activation in the face-selective regions (FFA & OFA). Contributions from LO and PPA were comparatively weaker and later. Note that commonality coefficients (ρ^2^) are often small in value (see for instance^41,45,46^), as there are likely other sources of variance not accounted for in the models. Therefore, their statistical significance is often considered more important than their magnitude. (**c**) fMRI ROI commonality in the Animal Study. Interestingly, the very earliest correspondence between RDMs arising from neural responses and movement trajectories shared overlap with representations arising in EVC, with later correspondences better accounted for by representations in anterior VTC. White outlines denote significant correlation clusters based on 10,000 permutations.

To inspect the functional contribution of different brain regions to the representational overlap observed between movement and M/EEG data, we performed a commonality analysis using pre-existing fMRI data (Fig. 5). Here we examined how much variance in the time-time movement-M/EEG correlation could be explained by each fMRI region of interest, when controlling for other regions. These analyses were conducted using the full RDMs while controlling for the task model in each study (i.e., the lower left panel for each study in Fig. 3). As seen in Fig. 5, the commonality results revealed that the strongest variance contributions came from the most functionally-relevant brain regions for the categorisation task in both studies.

In the Face Study, we tested the four regions of interest from^33^: the fusiform face area (FFA), the occipital face area (OFA), the lateral occipital cortex (LO), and the parahippocampal place area (PPA) (see RDMs in Fig. 5b, and “Methods” for details on ROI definitions). Although cluster-based permutation testing yielded significant and unique contributions of all four regions, the strongest contribution was found for FFA and then OFA, which are both critical brain regions whose activity dissociates strongly between faces and objects^40,41^.

In the Animal Study, we tested the three regions of interest from^31^: early visual cortex (EVC), posterior ventral occipitotemporal cortex (postVTC), and anterior ventral occipitotemporal cortex (antVTC) (see RDMs in Fig. 5c, and “Methods” for details on ROI definitions). We used the original fMRI RDMs from^31^ averaged across the two tasks (animacy and appearance), since these only moderately differed from one another, as was also the case for the corresponding EEG RDMs in^32^. Cluster based permutation testing revealed significant contribution to time-time movement-EEG correlations from all three regions, with the largest contribution arising for the most functionally-relevant brain region, antVTC, which is specialised in object categorisation^3,42^. This commonality arose relatively late (starting from about 140 and 350 ms in EEG and movement times, respectively). In contrast, a significant contribution from EVC corresponded to very early representational overlap between movement and EEG responses (starting from about 70 and 120 ms in EEG and movement times, respectively). This is in line with large and transient contribution of EVC to early EEG data (about 100 ms) reported in^32^, and more generally, with typical latencies observed along the visual hierarchy^1,2,6,43,44^. It is quite remarkable that we capture dissociations between such early visual responses, as it suggests that movement trajectories are capable of revealing early perceptual differences arising in primary visual regions and do so very rapidly after stimulus presentation.

## Discussion

The results here establish movement trajectories as an effective and accessible means of indexing dynamically unfolding neural representations, and that the representational structures captured in these movements are distinct from those reflected in conventional behavioural measures focused on the end-point of perceptual and decisional processes. Our findings highlight the relevance of the representational similarity analysis (RSA) framework to reveal informational content in movement trajectories and evaluate its correspondence with both behavioural and neuroimaging data, as well as with theoretical models. These findings open new avenues for investigating dynamic processes underlying human perception and cognition using cost-efficient, browser-based mouse-tracking paradigms.

Our results demonstrate that movement trajectories can provide a sensitive index of unfolding neural representations, even during the early stages of their formation. We provide strong evidence that categorisation movement trajectories are not ballistic, but rather dynamically integrate information contained in evolving visual representations. Our movement trajectories captured subtle time-varying representational differences between individual stimuli along two key dimensions within the human visual system—faces *vs.* objects and animals *vs.* objects. In our two studies, variability in online observers’ categorisation trajectories for face/animal, lookalike, and object stimuli showed meaningful correspondence with representational structure evident in unfolding M/EEG responses to these stimuli, even as early as 70 ms after stimulus presentation. Importantly, the central claim here is that variability in movement trajectories is meaningfully related to variation in the neural responses to the same stimuli. Notably, our results showed that the largest contributions to movement-M/EEG representational correlations came from the most functionally relevant brain regions for face/animal and object categorisation along the visual hierarchy^40,47^; namely, FFA and OFA (compared to LO and PPA) in the Face Study, and anterior VTC (compared to EVC and posterior VTC) in the Animal Study. The smaller but earlier contribution of EVC revealed in the Animal Study is particularly interesting, as it suggests that observers’ categorisation movements may even capture very early sensory processing at the beginning of the visual hierarchy. Together, these results demonstrate the suitability of movement trajectories as a proxy for unfolding visual representations in the brain, which opens new possibilities for disentangling how different stimulus features contribute to different processing stages underpinning human perception via cost-effective online experiments.

Beyond their relationship with neural measures, our results underscore the claim that movement trajectories themselves—even in relatively simple mouse-tracking paradigms as we use here^cf.^^48–50^—are a rich behavioural measure that can afford insight into the dynamics of human perception and cognition that conventional discrete measures cannot provide^7,10,51^. Here, we found that the timepoint-by-timepoint representations indexed via movement trajectories—in particular, the earlier and more subtle image-wise representations—were distinct from those captured in discrete behavioural indices that represent the culmination of many perceptual and decisional processes. In both studies, movement-M/EEG representational overlap was not accounted for by variation in the overall response speed (i.e., movement execution time). Neither was it explained by explicit face-likeness ratings (obtained by^33^) in the Face Study, or by explicit stimulus similarity judgements (obtained by^31^) in the Animal Study. Of particular relevance here is the fact that the early stages of our movement trajectories successfully captured the categorical ambiguity of lookalike stimuli in the Animal Study—a finding which was observed in the neural response to these images^32^, but missing in the explicit similarity judgement data^31^. These results provide strong evidence supporting the claim that movement trajectories are ideally suited to capture intermediate representations in perceptual and cognitive processes^10,52^. Our results confirm the ability of movement trajectories to index implicit and competitive cognitive processes that are otherwise blurred or already resolved by the time discrete explicit behavioural measures are obtained (e.g., button presses, ratings, and questionnaires)^11–15,53–55^.

Our findings underscore RSA as a powerful framework with which to reveal dynamically changing information content in movement trajectories. Timepoint-by-timepoint representational structures extracted from movement trajectories via RDMs enabled us to track the evolving information content reflected in observers’ categorisation movements from immediately after stimulus presentation through to their final categorisation response. Correlating these representational structures with different theoretical models revealed the timecourse over which initially ambiguous category representations for lookalike stimuli are resolved. Abstracting away from measurement units also enabled us to evaluate the correspondence between information content reflected in behavioural and neuroimaging measures with very distinct timescales (i.e., M/EEG, fMRI). This work follows from prior studies that have used RSA to combine mouse-tracking data with human neuroimaging data, which have successfully revealed the relevance of specific brain areas to stereotype biases in face perception^56^, culture-specific perception of facial emotion/contextual associations^57^, and social biases^58^. The current work goes a step further, leveraging RSA to quantitatively compare the dynamics of representational structures carried by movement and M/EEG data through a temporal generalisation approach.

This work provides compelling proof-of-concept that movement trajectories can be adopted as a sensitive index of dynamically evolving visual representations that have previously only been accessible via time-resolved neuroimaging methods (M/EEG). Where we have focused on the capacity of movement data to capture meaningful variation in neural representations of faces, animals, and objects, our RSA-based approach has broad relevance for the study of dynamic processes in general, as it can be customised to probe the evolving representational geometry of any kind of stimulus about which observers can make a judgement. This could range from perceptual estimations of low-level stimulus features (e.g., Gabor orientation or spatial frequency) to semantic or affective judgements of images or words (e.g., “Is this item found indoors or outdoors?”, “Is this item positive or negative?”). By evaluating the correspondence between movement trajectories on a range of different tasks with both theoretical models and with the increasing number of publicly available EEG, MEG, ECoG, fMRI, fNIRS, EMG and eye-tracking datasets, we believe the approach holds enormous potential for answering diverse questions about human perception and cognition in future research.

## Perspectives and limitations

While our results demonstrate the great potential of movement trajectories for studying visual representations, there are still outstanding questions that can be addressed in future work. Our results show a meaningful relationship between the representational structures in neuroimaging and movement data but the limits to which this relationship holds is currently not fully explored. For example, how well do movement trajectories capture the very early stages of neural representations? Are representations captured in neuroimaging and movement data exactly the same? What is the delay after which neural representations are observed in movement trajectories? Is this delay constant? These are important questions that future research will need to address to better understand the exact relationship between neural and movement representational structures. Furthermore, beyond early automatic neural representations tested here, future research will also benefit from exploring later and more task-related dynamic processes, such as those involved in decision-making. In addition, it will also be important to compare different movement trajectory tasks, as well as classic decision-making models such as evidence accumulation models^59^ to determine their contribution to movement-M/EEG correlations. Distinguishing neural representations in correct and incorrect trials would also be of interest to fully understand what information related to early automatic neural representations and later decisional processes is exactly captured in movement trajectories.

## Conclusion

This work highlights critical advantages of movement trajectories for studying dynamic brain processes. They are as time- and effort-efficient as most explicit behavioural measures, while providing richer information, specifically the timecourse of covert perceptual and cognitive processes. Combined with RSA, online and open-access resources, movement trajectories offer a powerful, cost-effective, and widely accessible method to advance human behavioural and cognitive science.

## Methods

### Participants

Participants for the online mouse-tracking experiments were first-year psychology students from Western Sydney University, recruited via the SONA platform in exchange for course credit. We recorded data from 268 participants in the Face Study and 253 participants in the Animal Study. We removed trials on which less than 100 ms of cursor datapoints were recorded, or did not move at least 50 pixels. We further removed any participants with an overall accuracy rate below 70%, where a trial was counted as ‘correct’ if the final cursor position was on the same side of the screen as the correct response option for that trial (35% of participants removed in the Face Study, 27% of participants removed in the Animal Study). The final number of datasets retained for further analysis was 174 in the Face Study (141 females, age = 23.17 ± 7.94, right-handed = 158, native English speakers = 124) and 185 in the Animal Study (137 females, age = 22.71 ± 8.60, right-handed = 171, native English speakers = 138).

The study was approved by the Human Research Ethics Committee of Western Sydney University (H14498). All participants provided written informed consent prior to the study. The experiment was performed in accordance with the Declaration of Helsinki and relevant guidelines and regulations for research involving human research participants.

### Stimuli

The Face Study used 96 naturalistic colour images from^33^, consisting of 32 face, 32 lookalike (illusory faces), and 32 object stimuli, all unsegmented from their surrounding backgrounds. Objects and lookalike stimuli were individually matched (see Fig. 1a, left column). The Animal Study used the 27 greyscale segmented images from^31^ and^32^, consisting of 9 animal, 9 lookalike, and 9 object stimuli. These stimuli were individually matched across all three categories (see Fig. 1a).

### Procedure

The online mouse-tracking categorisation experiments were hosted on Pavlovia^60^ and written in JavaScript (jsPsych 6 libraries^61^) based on publicly available code^62^ (https://github.com/mahiluthra/mousetracking_experiment). The experiment ran locally in a web browser on the participant’s own computer^35^. Each trial began with a central fixation cross and a “Next” button at the bottom of the screen that the participant had to click to initiate the trials sequence. This ensured the cursor was repositioned at the bottom of the screen at the beginning of every trial (see Fig. 1b). After the trial sequence began, the fixation cross remained present for 200 ms to promote movement readiness, before the target image appeared at fixation with two response boxes in the upper left and right corners of the screen. One of them contained the word “OBJECT” and the other “FACE” (Study 1) or “ANIMAL” (Study 2). The position of the two response boxes was swapped halfway through the experiment (i.e., after two blocks), with the initial position of response boxes counterbalanced across participants to avoid right/left movement biases. Participants had 800 ms to move the cursor to the correct response box, after which the trial ended automatically. Clicking on one of the response boxes also ended the trial. Cursor position was recorded throughout the 800 ms after trial initiation at the maximum sample rate of the local system (1000 Hz). To equalise the probability of object and face/animal responses, we augmented the original stimulus sets with an additional 32 faces in Study 1 and 9 animals in Study 2 (additional stimuli excluded from analyses). All 128/36 images appeared in each block; there were four blocks in total, and participants could take self-paced rest breaks between each block as necessary.

### Mouse-tracking movement trajectory pre-processing

We analysed mouse-tracking data in MATLAB using custom-developed in-house scripts (https://osf.io/q3hbp/). We considered all completed trials for analyses (both correct and incorrect categorisations) as they jointly represent the unfolding of categorical representations. Empty values were replaced with NaNs at the beginning of the mouse-tracking recordings (due to late movement onsets) and the end (due to response box clicks before 800 ms). We then linearly interpolated the data to 1 ms intervals from 1 to 800 ms. For each of the 96 and 27 images in the Face Study and Animal Study respectively, we averaged the horizontal and vertical position data at each timepoint, firstly across trials for each participant (4 trials per image, if all completed), and then across participants.

### Representational similarity analysis (RSA)

We used RSA to relate the movement trajectory data to other experimental data via representational (dis)similarity matrices (RDMs). Calculated as the pairwise dissimilarity between all stimuli, RDMs (96 × 96 and 27 × 27 in the Face and Animal Study, respectively) serve to abstract data away from original measurement units to capture its information content. RSA enabled us to test the representational overlap between movement data, M/EEG data, fMRI data, theoretical models, and control behavioural models.

### Movement RDMs

We calculated an RDM for every timepoint following stimulus presentation until the end of the trial (i.e., 800 ms), computed as the absolute difference in cursor horizontal position between each pair of stimuli. Since the horizontal axis is the relevant decision axis for our task, the resulting RDM provided a time-varying index of face/animal *vs.* object categorisation. In line with previous research using online data^35^, RDMs were computed on cursor horizontal position after averaging across all participants to compensate for a high level of noise inherent to online data with fewer trials.

### M/EEG RDMs

For the Face Study, we used individual participant MEG RDMs between 0 and 500 ms from stimulus onset, taken from^33^. MEG RDMs were obtained from 22 participants using a 160-channel whole-head KIT MEG system. The MEG task consisted of the presentation (200 ms) of the 96 visual stimuli (24 repeats of each stimulus). In each trial, images were tilted by 3° (left or right), and participants had to report the tilt direction. MEG data were down-sampled to 200 Hz, and PCA was applied for dimensionality reduction (retaining PCs explaining 99% of variance). MEG RDMs were constructed by taking 1-correlation (Spearman) between the MEG activation patterns for each pair of stimuli at each time point (i.e., every 5 ms).

For the Animal Study, we used individual participant EEG RDMs between 0 and 500 ms from stimulus onset, taken from^32^. EEG data were obtained from 30 participants using a 128-channel active electrode system. Participants performed two tasks in alternating blocks, responding yes/no to either: “Does this image depict a living animal?” (Animacy task) or “Does this image *look* like an animal?” (Appearance task). EEG data were down-sampled to 250 Hz, high-pass filtered at 2 Hz, and 50 Hz notch filtered while artifacts were removed using ICA. EEG RDMs were obtained using temporal searchlight decoding analyses. At each timepoint, an LDA classifier was trained and tested on discriminating between activation patterns associated with each pair of images using leaving-one-run-out cross-validation procedure. RDMs were constructed using the decoding accuracy as an index of neural dissimilarity at every timepoint (i.e., every 4 ms), separately for each task, and for the pooled data of both tasks. Here we used the latter RDM series since visual object representations emerging in the brain milliseconds after stimulus presentation are largely automatic and independent of the behavioural task used, with key object categories being observed in neuroimaging data whether or not those are explicitly tested behaviourally^6^. The (non-task specific) variance targeted here (as often in the object recognition literature) was therefore more objectively captured by combining the two tasks.

### fMRI RDMs

For the Face Study, we used neuroimaging data from^33^. Functional MRI recordings from 16 participants were acquired using a 3T Siemens Verio MRI scanner and a 32-channel head coil. A 2D T2*-weighted EPI acquisition sequence was used: TR = 2.5 s, TE = 32 ms, FA = 80°, voxel size: 2.8 × 2.8 × 2.8 mm. The fMRI task was analogous to the MEG task described above, save that stimuli were presented for 300 ms followed by a grey screen to complete a 4 s trial. All stimuli were shown once per run, and each participant completed 7 runs. Data were slice-time corrected and motion-corrected using AFNI. An independent functional localiser experiment using a different set of images was performed to define the category-selective regions: FFA, OFA, LO and PPA. fMRI RDMs were built by taking 1-correlation (Spearman) between the BOLD signal for each pair of stimuli (96 × 96) in each of the four category-selective areas.

For the Animal Study, we used neuroimaging data from^31^. Functional MRI recordings from 17 participants were acquired using a 3T Philips scanner with a 32-channel head coil. Functional images were recorded using a T2*-weighted EPI with acquisition parameters: TR = 2 s, TE = 30 ms, FA = 90°, voxel size: 3 × 3 × 3 mm. Participants performed animacy and animal appearance tasks for 12 runs each (task order counterbalanced across participants). fMRI RDMs were built by taking condition-wise parameter estimates per participant and image runs. Correlations (Pearson’s r) were computed using bootstrap sampling (for further details see^31^). Briefly, in each iteration (*n* = 100), data were partitioned into random 2 subsets and the 27 × 27 correlation matrices were averaged across iterations and converted into dissimilarity matrices (1-Pearson’s r) for each task (animacy and appearance). In our analyses, we averaged RDMs for both tasks, as done in^32^ since these only moderately differed from one another.

### Theoretical models

We used two theoretical models that differentially assigned the ambiguous stimuli in our experiments to either their true category or the category for which they could be confused (see Fig. 2). The Face/Animal model represented the ground truth for our categorisation tasks (i.e., ambiguous stimuli grouped with Objects). The Lookalike Model represented the perceptual similarity between stimuli (i.e., ambiguous stimuli grouped with Faces/Animals). These binary theoretical models were correlated (Spearman ρ) with both the movement and M/EEG RDM series, yielding a time-resolved correlation for each model with each dataset.

### Control behavioural RDMs

To clarify the degree to which correlations between movement and neural RDMs could be explained by discrete/explicit behavioural measures, we built two behavioural control models for each Study (see Fig. 3a). For both studies, we constructed an Execution Time RDM, calculated as the pairwise differences in the time taken in milliseconds to reach the categorisation response box, therefore, using only trials where a response box was reached (87.4 and 85.5% in the Face Study and Animal Study, respectively). For the Face Study, we used the Face Ratings RDM from^33^ that was based on pairwise dissimilarities in face-likeness ratings (“Rate how easily you can see a face in this image on a scale of 0–10”). For the Animal Study, we used the Similarity RDM from^31^ that was based on participants’ behavioural judgements of image similarity (observers saw all the test images simultaneously and had to “arrange the objects according to how similar they are”, see^31^ for details).

### Movement-M/EEG time-time representational correlation

We calculated the (Spearman) correlation between time-resolved movement and M/EEG RDMs at every timepoint combination, resulting in 500 ms (M/EEG time) × 800 ms (movement time) maps presented in Figs. 3 and 4. This temporal generalisation approach (see^63^ for a review) allowed us to identify sustained and repeated overlap between movement and M/EEG representational structures and latencies along each dimension. We calculated time-time correlation on the full RDM (96 × 96 and 27 × 27 in the Face Study and Animal Study, respectively) to reflect representations of the full stimulus set (see Fig. 3) and on specific subsets of the RDMs (32 × 32 and 9 × 9) to focus on image-wise representations (see Fig. 4). We used partial correlation with the Task Model (i.e., Face/Animal) as covariate to control for representational structures in the full RDM directly resulting from the face/animal *vs.* object mouse-tracking categorisation (e.g., go left for FACE, go right for OBJECT). Partial correlation was also used with control behavioural models as covariates to control for representational similarity explained by more conventional behavioural measures.

### Commonality analysis

We used commonality analysis to test the contributions of each fMRI ROI to time-time movement-M/EEG correlations^45,64^—FFA, OFA, LO and PPA in the Face Study, and EVC, posterior VTC, and anterior VTC in the Animal Study. This method has successfully been used in conjunction with RSA to compare how different predictors in the form of neuroimaging methods, models, and tasks explain shared variance^41,45,46,65^. fMRI RDMs are not time-resolved, since BOLD responses are very slow. Thus, the single RDM for each fMRI ROI was used for all movement-M/EEG correlation timepoint combinations (all fMRI RDMs present in Fig. 5). For a given ROI_i_, the commonality was calculated as the difference between the movement-M/EEG partial correlation controlling for all ROIs except the given ROI_i_ (Mov, M/EEG, ROI_i+1_, ROI_i+2_, ROI_i+3_), and the movement-M/EEG partial correlation controlling for all ROI including the given ROI_i_ (Mov, ROI_i+1_, ROI_i+2_, ROI_i+3_, ROI_i+4_):

$$\begin{aligned} C\left( {Mov, \, M/EEG, \, ROI_{i} } \right) & = R^{2} \left( {Mov, \, M/EEG, \, ROI_{i + 1} , \, ROI_{i + 2} , \, ROI_{i + 3} } \right) \, \hfill \\ & \quad - R^{2} \left( {Mov, \, M/EEG, \, ROI_{i} , \, ROI_{i + 1} , \, ROI_{i + 2} , \, ROI_{i + 3} } \right) \hfill \\ \end{aligned}$$

The statistical significance of commonality coefficients (ρ^2^) is often considered more important than their magnitude. Commonality coefficients often have small values^41,45,46^, as other sources of variance are not accounted for in the models.

### Statistics

Confidence intervals for the correlations of movement and M/EEG RDMs with the theoretical models in Fig. 2 were estimated using jackknife resampling. We selected participants with random replacement from the sample and recalculated the group-level correlations 10,000 times. These correlations values and their difference between the two models were used to estimate one-tailed significant differences from 0 at α = 0.05 (blue and red dots) and two-tailed significant differences from 0 at α = 0.05 (grey dots), respectively. For time-time correlations between movement and M/EEG RDMs (Figs. 3, 4, 5), we used cluster-based permutation testing to control for multiple comparisons^66^. For each iteration (*n* = 10,000), we sign-permuted the time-time maps at the participant level and tested each time-time point against 0 using one-sample *t*-test. We set a cluster threshold at α = 0.05, and took the largest sum of the *t*-values from each cluster value to construct the null distribution. Clusters with a larger cluster-sum statistic than 95% of the null distribution were deemed significant. For all statistical tests, the M/EEG participants’ data served as the source of variance rather than the movement data. The latter was only ever considered at the group-average level, since online data that tend to be noisy at the individual participant level become highly reliable when averaged across many participants^35^.

## Data Availability

Mouse-tracking data are available at: https://osf.io/q3hbp/.

## Code availability

MATLAB code to produce all results and figures are available at: https://osf.io/q3hbp/.
